# The Missing Link: How New Institutions Could Improve What We Read Online

**DOI:** 10.1093/ojls/gqag013

**Published:** 2026-04-15

**Authors:** Martin Husovec

**Keywords:** platform regulation, user empowerment, public sphere, content governance, algorithmic curation

## Abstract

While the EU’s Digital Services Act and the UK’s Online Safety Act represent a major shift in Internet regulation, they suffer from a critical structural gap. These frameworks focus primarily on ‘defensive empowerment’—enabling users to block or report unwanted content while neglecting constructive empowerment—the ability of users to decide what information they wish to see. The article argues that without collective institutions to signal content quality, users cannot assess quality beforehand, creating a market failure that pushes out expensive, high-quality content in favour of cheap, sensationalist, algorithm-driven content. To bridge this gap, the article proposes the creation of trusted content creators: voluntary, self-governing groups of like-minded content creators that certify content quality through cross-platform labels. By mandating that platforms allow users to filter their feeds through these external quality signals, the law can decentre the role of algorithmic curation and create new markets.

## 1. Introduction

Online platforms usually aim to ‘organise the world’s information’ (Google), ‘bring the world closer together’ (Meta) or some combination of the two. While new means of information exchange have benefited humanity tremendously, facilitating it without editors and globally has brought us many challenges today, ranging from ever-present unreliable information to novel forms of abusive behaviour and new tools capable of undermining democratic processes. Navigating online information has been made especially difficult by the fact that there are no quality-oriented non-state institutions that could assist users on the Internet’s terms, that is, at its speed and scale.

This article offers a way forward. It is organised as follows. This section examines the structural transformation of the public sphere from mass media to networked platforms, explaining how the loss of professional filters creates information asymmetry that drives out high-quality content. Section 2 explains the evolution of platform regulation, its limits and potential pitfalls. Section 3 shows that focus on enforcing the legality of content and design, while important, fails to help users to navigate content quality due to missing curatorial institutions. Section 4 presents the detailed proposal for trusted content creators (TCCs)—voluntary certification groups that label content and enable user-controlled curation across platforms. Section 5 concludes by conceptualising TCCs as an institutional infrastructure that disperses communicative power and facilitates democratic knowledge aggregation.

### A. Structural Changes of the Public Sphere

A functioning public sphere is essential for the ability of democracies to solve collective problems.^1^ The quality of democratic decision making depends on the quality of public deliberation, which in turn relies on the quality of information informing that deliberation.^2^ The institutions that sustain public discourse are therefore not optional but a constitutional necessity.^3^

The public sphere has changed a lot over the past two centuries. In his seminal work, Habermas first described a shift from the participatory culture of coffee houses, salons and civic societies towards the unidirectional mass media of the 20th century, in which citizens became passive consumers of information.^4^ This transformation has been attributed to the economics of long-distance mass media, which is characterised by high entry costs and centralised production.^5^

The rise of the Internet revolutionised information distribution by enabling anyone to reach large audiences and share their expertise or opinions. This development weakened traditional information and attention gatekeepers,^6^ such as newspapers and broadcasters. The decentralisation of content production democratised authorship and drastically reduced distribution costs.^7^ Habermas identified this as another major transformation of the public sphere.^8^

In the latest transition from the mass-mediated public sphere of the 20th century to the networked public sphere of the 21st,^9^ media organisations have disaggregated into individual content creators—bloggers, independent journalists, podcasters and vloggers. This change has made the public sphere more participatory again. However, influence has shifted from traditional institutions to individuals, who have become the new units of content production.^10^ As DiResta and Kleinfeld observe, the appeal of individual content creators now lies in their perceived proximity to audiences.^11^

Influencers win attention and legitimacy via authenticity, relatability, and direct audience connection. Institutions and experts, by contrast, generally continue to signal credibility via authority: facts presented alongside credentialling markers such as educational degrees, institutional brands, or peer review.

This shift made observance of traditional quality standards secondary, leading Habermas to call it the trend of ‘deprofessionalisation’.^12^ Platforms have become the key spaces where the networked public sphere is cultivated and incentivised. As research on technology and architecture has shown, spaces for deliberation shape human interaction and, consequently, social and political order.^13^ Habermas cautioned that the latest transformation causes society to lose ‘professional filters’^14^ that ensure the ‘reliability, quality and general relevance of public contributions’.^15^

The ecosystem incentives are gradually undermining other institutions that helped us to produce reliable information, thanks to content quality standards. Editorial content is losing ground to content of lesser quality, and the business model of legacy media is being severely weakened by superior advertising services offered by platforms. Media are ever more dependent on the distribution by platforms.^16^ Despite the extension of networked public, thus, there is a risk of losing expensively produced reliable information, which is a by-product of applying quality standards.

The loss of reliable information represents a huge social cost for everyone in the long run. Societies function better if they are based on better information.^17^ Reliable factual information empowers people to make good social, economic and political decisions.^18^ It promotes good governance and builds social trust and civic participation.^19^ It also increases productivity, and thus enhances well-being.

### B. Curatorial Tutelage of Platforms

In the public discussion, *platform design* has become the centre of conversations about how to re-establish mechanisms to maintain content quality standards in user-generated content environments. The focus is largely on algorithms that lie at the core of the networked public sphere. Algorithms power recommender systems that determine the visibility and reach of user-generated content.

Designed around engagement metrics, recommender systems respond to user behaviour, but only to revealed preferences (clicks, time spent, shares) about content quality. This creates a principal-agent problem: even if users want reliable information, algorithms optimise for immediate engagement,^20^ which low-quality content often generates more effectively. Users cannot express preferences for unobservable qualities like ‘fact-checked’ or ‘editorially independent’, especially for unknown sources of information, within the current platform architecture. The problem intensifies as platforms shift from social graphs (explicit follows) to interest graphs (algorithmic inference), reducing users’ ability to curate through social connections. Today, only 7% of content on Instagram and 17% of content on Facebook comes from connections, such as friends or follows.^21^ This does not mean algorithms are necessarily misaligned with all user interests, but rather that they cannot capture the full range of what users value.

Some conclude from this that platform profit motives that inform the design of such algorithms cannot be aligned with broader societal interests. Couldry, for instance, identifies a problem in that such algorithms are being operated by for-profit companies in their commercial products that are powered by advertising.^22^ However, the evolution of legacy media demonstrates that such a diagnosis is too sweeping.

Alignment of profit maximisation of commercial content curators and broader societal interests, while difficult, is not impossible. After all, Edmund Burke’s characterisation of media as the fourth estate in the times of the French Revolution was validated by their growing importance in liberal democracies ever since. Moreover, as shown by James Hamilton, modern journalistic quality standards and editorial independence have emerged as a commercial product because independence, as opposed to political partisanship, appealed to broader readership and thus enlarged the profit of newspaper owners.^23^

While news organisations are driven by profit maximisation, they strongly respond to consumer preferences, and their commercial interests often align with the public interest.^24^ Competition among media organisations advances society’s interests by countering government influence and encouraging timely and accurate reporting through competitive pressure.^25^

Arguably, the race to the bottom in terms of content quality online is rather caused by the environment of choices that users face.^26^ Applying Akerlof’s work on information asymmetry^27^ to the network public sphere reveals that online experiences are defined by the fact that users usually discover they have been tricked into watching or reading low-quality, false and low-cost content *after* they have already spent their attention.^28^ Users’ inability to verify quality, including veracity of information, before they consume new content creates a structural market failure that systematically drives out costly, high-quality production in favour of cheap, sensationalist or clickbait content. Akerlof’s work shows that the policy response to such market failures should be to strengthen signals of trust to facilitate that users and producers alike invest in high-quality products.

Approached from this perspective, the key problem is not so much the link of platform recommender systems with profit-maximising entities as the inability of users to exercise informed curatorial choice due to a lack of credible signals of quality of content. The stronger the algorithmic curation, the greater the barriers to audience reach that content creators face if the production qualities of their content differ from those prioritised by the platforms’ curatorial algorithm. Likewise, the stronger the algorithmic curation, the more difficult users find it to tailor or optimise their online experience to interests and preferences that are not reflected in such an algorithm.

Whereas earlier platform architectures relied on follows and friendships to construct ‘social graphs’ that mediated direct content distribution, platforms are increasingly abandoning this model in favour of an ‘interest graph’ that algorithmically infers our presumed interests.^29^ In response to competition from TikTok, for example, Facebook and Instagram now prioritise algorithmic recommendations over users’ explicit social connections or expressed preferences.^30^ On the marketplaces, too, algorithmic systems increasingly decisively curate what reaches shoppers.^31^ The absence of curatorial choice allows platforms to ‘monopolise’ the user’s attention within the service because users can only either leave the platforms entirely, which entails large opportunity costs, or remain. While such private power often does not amount to a typical monopoly under competition law, laws like the EU’s Digital Markets Act and the UKs Digital Markets, Competition and Consumers Act recognise it as a problem because, for creators who are businesses, the platform’s algorithm is an important gateway to consumers.^32^

**Figure 1.**
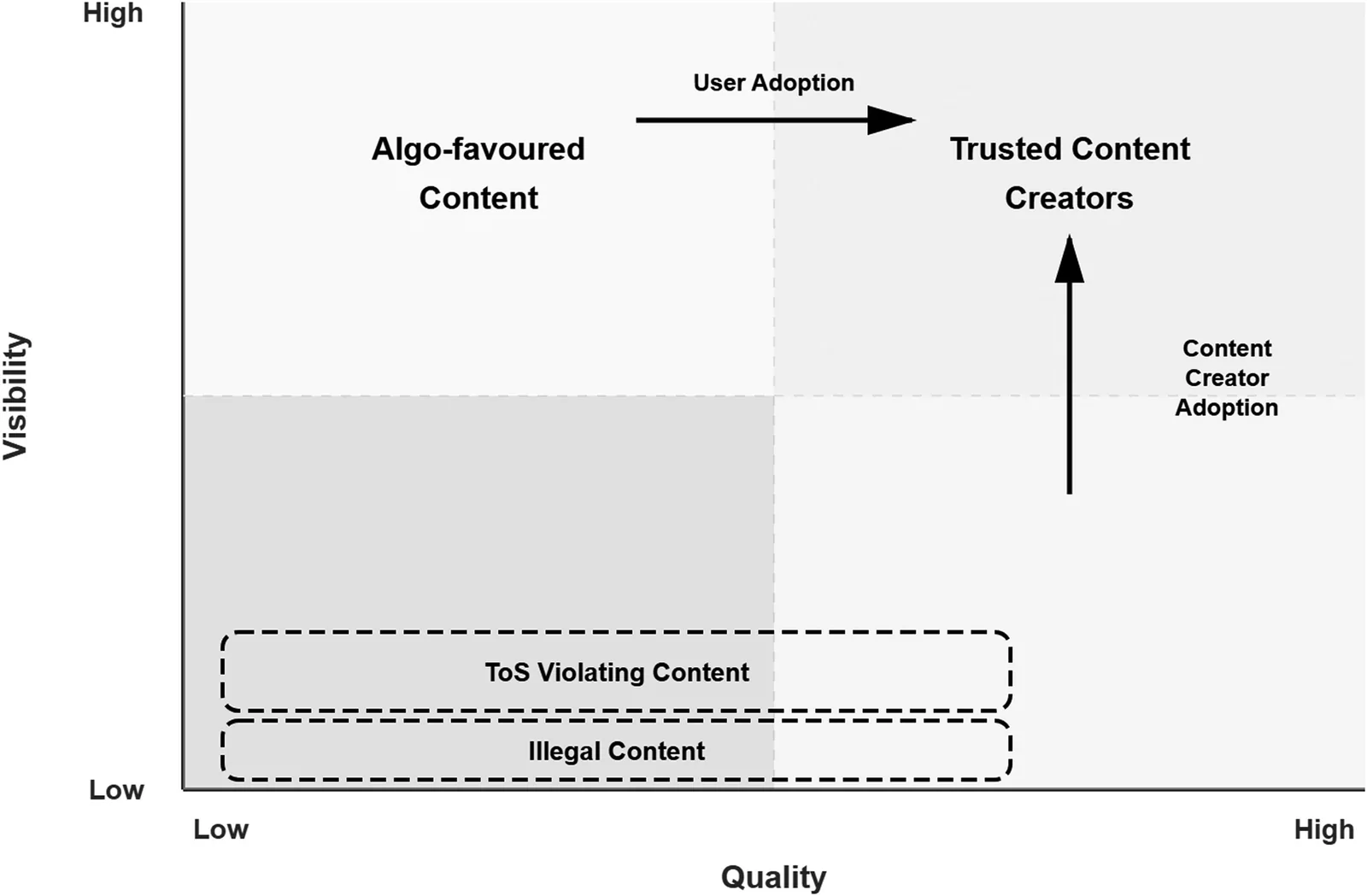
User adoption of TCC labels shifts the content consumption to high-quality sector, as shown by the horizontal arrow, which in turns attracts content creators to join or form new TCCs as show by the vertical arrow.

Therefore, the central challenge in the contemporary networked public sphere is restoring user agency in content curation. But to meaningfully restore user agency, we first need to solve the information asymmetry relating to the quality of content. My proposal is to do so through new institutional mechanisms that act as certification points for quality. While users can already today navigate uncertainty by following brands of content creators they trust, this does not help when discovering new content. Platform algorithms also increasingly unbundle content by showing individual articles, often stripped of the context of the publisher’s reputation.^33^ Moreover, individual brands rarely act as full guarantees because some qualities, such as veracity, are hard to observe for users even after consuming content. We need a user-driven system that shifts the visibility from low-quality content to high-quality content (see Figure 1).

### C. The Structural Gap in Institutions

Many of the current scholarly and policy works are focusing on how to redesign the ranking within recommender systems.^34^ A lot of thought is being put into how to build alternative systems of prioritising or filtering of user-generated information that could better align with society’s goals.

If such proposals become the only strategy for addressing the problem, they could cement the role of platforms’ systems as the centre of gravity for quality selection.^35^ They could put too much faith in the detailed central planning of design and values that ought to underlie commercial communications infrastructure. Such planning, as extensively explained by Hayek, would inevitably fail.^36^ Any detailed plan, even one that was made with perfect information and the best of intentions, would become outdated and thus out of sync with society’s dispersed knowledge in a few months’ time. That is not to say that governmental design mandates are not necessary. On the contrary, user empowerment against private power is often impossible without governmental intervention. However, the more detailed the mandates, the more likely they are to fail.

In other words, the problem with such design-based solutions to content quality is not that we do not need ‘professional filters’, or that platforms cannot implement them, but that *we first need to find quality standards* through deliberation between users and content creators and then *keep revisiting* them. Quality standards are dynamic, and often not known *ex ante*. TCCs are thus curatorial institutions to find those standards continuously. They should become ‘professional filters’ that certify content qualities and be operationalised as user-controlled lenses through which user-generated content is selected and consumed within platforms. As explained later, some governmental design mandates are necessary to make it happen.

Directionally, rather than cementing the role of platforms, the law should decentre functions of quality selection from one central commercial algorithm to a dispersed set of non-state institutions that aggregate and codify deliberation on quality standards among their members. Under this conception, the platform companies would still be expected to assist. However, the main role of companies would be to put in place the basic infrastructure for deliberation about quality standards by content creators and users, and not to become the chief architects of such quality standards themselves.

Today, even if companies were ready to rely more on exogenous institutions to sort the quality of content, they would often have a hard time finding the appropriate ones. Appropriate institutions are largely missing, and this creates a vicious circle: if institutions do not exist,^37^ they cannot be woven into the products to improve them. Non-state institutions, especially those that would enable deliberation around content standards, are thus the *missing link* between decentralised user-generated content and the creation of social order. Even though communication tools connect us, paradoxically, they at present do not link users and content creators in ways that would facilitate their deliberation about what is trustworthy and what is not. The communication link to do so and a place to signal agreement on content standards are missing.

Meta’s recent controversy is illustrative. Meta used, without authorisation, a trusted certificate mark, ‘PG-13’, to empower parents on the platform.^38^ The PG-13 mark was developed by the film industry to label whether films are inappropriate for specific age groups. Had MPA been allowed to label the content of its members (or external creators) that conforms to such a standard, Meta could have simply directed parents to choose to prioritise those labels in the user settings.

User-generated content for now continues to exist in a social and political vacuum, fully reliant on signals generated within services, such as likes, follows, views or engagement. However, the fact that something garners attention or emotional approval has little to do with whether it breeds trust. This vacuum thus results in calls by users to either market forces, that is, platforms, or state power, that is, legislatures or regulators, to fill it with measures that satisfy opposing viewpoints by banning or allowing content.

The EU and the UK recently introduced novel regulatory regimes to deal with some of these challenges. The EU’s Digital Services Act (DSA) and the UK’s Online Safety Act (OSA), however, also do little about the inadequacy of our content-related institutions. They do not challenge the thinking that, on the collective level, there are only two legitimate levers to better organise the new public sphere. Either we must expect the state to intervene more by mandating that some content or behaviour becomes legally restricted in specific jurisdictions, or we must expect the platforms to intervene by banning content or behaviour in their terms of conditions, or by ‘deamplifying’ it across the services. While the laws pioneer granting more individual empowerment to users, that empowerment is mostly of a defensive kind; it helps individuals to avoid seeing what they do not want to see. Empowerment of a constructive kind, which would help them to see more of what they want to see, is largely missing.

The primary reason is that the key focus is on what platforms should do to better enforce existing laws online. Regulations thus miss that there is an equally big need for users to better deliberate about appropriate standards for content. The OSA and DSA fail to contemplate new institutions that could complement the efforts of states or platforms to better organise information by putting content creators and users in charge. This oversight has considerable practical consequences. In the absence of a third choice that would allow people to opt in to standards they recognise as trustworthy, the pressure mounts on advocating that either states or platforms ban or hide content centrally for everyone.

Unlike earlier literature, this article thus proposes an institutional supply-side solution: creating new, portable signalling mechanisms that restore the ability of users to distinguish ‘quality’ *ex ante*, effectively converting online content, or its premium subset, into a product of verifiable qualities. The article proposes legal incentives to develop new collective institutions—so-called trusted content creators—that can complement the ways in which the state and platforms order the information online.^39^ Such institutions should inject more empowerment into how we organise information online, allow users to express support for different ways of ordering, thus facilitating consensus building, and finally, give them the option to customise their user experience accordingly.

### D. Trusted Content Creators—A Brief Outline

Before proceeding any further, it is necessary to briefly outline the solution proposed in this article. The discussion of various features of the system will be addressed in the final section.

TCCs are voluntary, self-governing associations of content creators who collectively define and enforce standards of content-production quality and signal compliance through a portable label that operates across platforms. Each TCC establishes its own rules of common practice, reviews prospective members and monitors adherence through peer-based oversight and sanctions. The TCC label functions as a cross-platform certification mark, allowing users, platforms and regulators to recognise creators who meet defined quality standards. TCCs thus constitute a non-state, bottom-up institutional mechanism for producing credible and portable indicators of quality in the networked public sphere.

**Figure 2.**
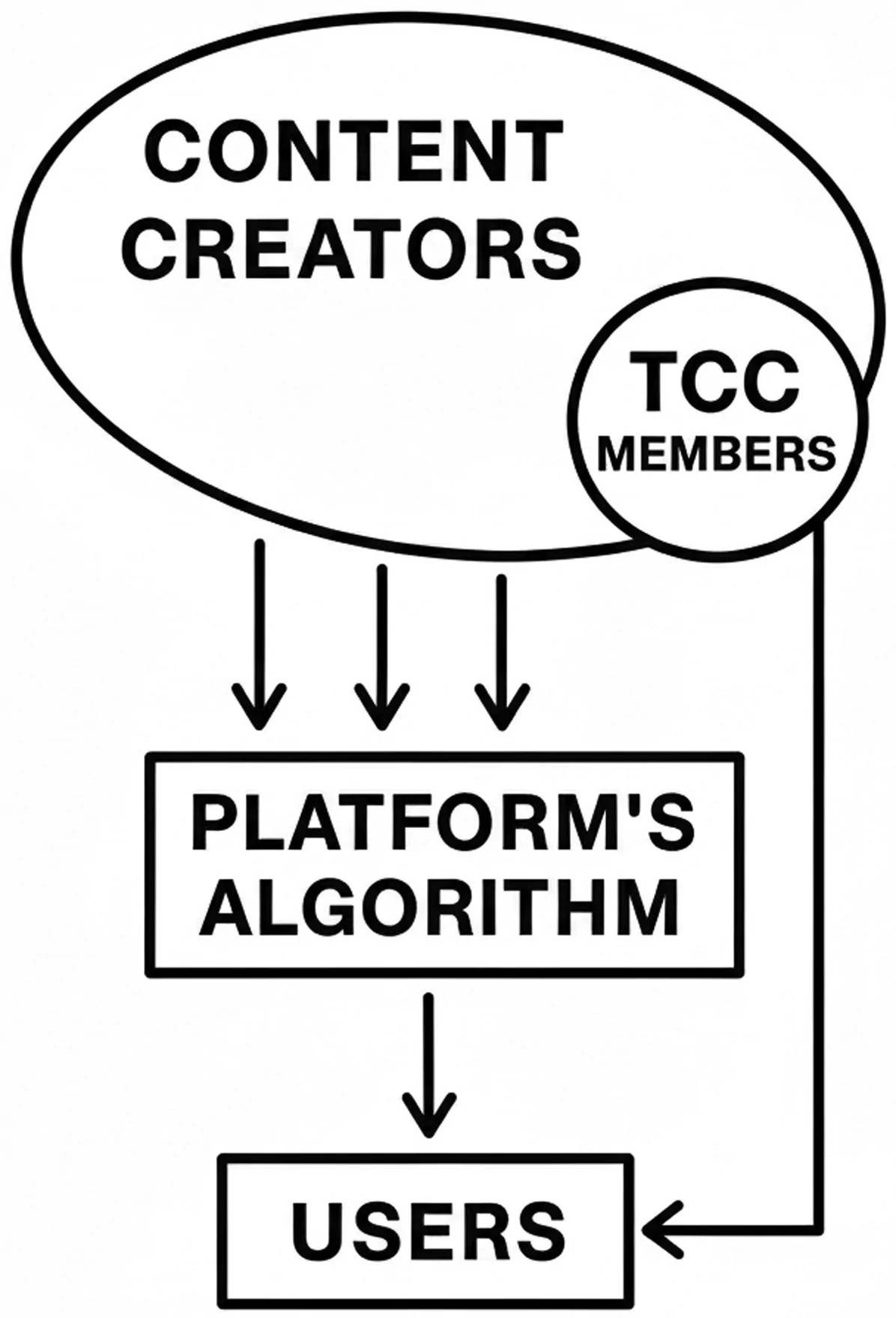
The direct communication channel between TCC members and users, as shown by arrows, bypasses platform's algorithmic curation that traditionally mediates content consumption by users.

TCC labels create a new meta-communication channel between the collectives of content creators and individual users. Unlike regular forms of platform engagement, such as follows or friendships, the labels create a new possibility to express user preferences based on quality standards which are currently unobservable and inexpressible. The labels allow content creators to distinguish their content of premium quality and users to reward it with more attention, thanks to newly created signals of quality. Users can reward the quality labels by subscribing to them and deciding to prioritise content corresponding to TCC labels in their user settings. This voting by users bypasses the platforms’ algorithmic curation mechanism (see Figure 2).

TCC labels would operate alongside, and not as a replacement for, platform algorithms. When users subscribe to a TCC label in their settings, the platform’s recommender system incorporates them as a preference signal. The legislatures could mandate channels for TCC-mediated content through separate feeds or adjustments of the existing feeds, or both in parallel. Regardless of the modality chosen, the platforms retain control over their algorithms but lose the ability to force users to consume their algorithmic choice on a take-it-or-leave-it basis.

For example, if a user subscribes to ‘Science Communicators TCC’, ‘Non-partisan Journalists TCC’, ‘War Journalists TCC’ and ‘Fair Product Reviewers TCC’, all separate groups of content creators with their own set of rules about how to create content, the user would be guaranteed to see such content in the separate feed or have a stronger likelihood of seeing it in the existing feeds. Users adopt such labels because they trust the quality of the content of some TCC members, through which they start encountering more content of the same quality from other members of the same TCC. Users adopting TCC labels as lenses to content discovery would either completely opt out of the algorithmic choice of platforms, while continuing to use those platforms, or heavily reduce their exposure to content outside of TCC groups of their own preference. The more users adopt TCC labels as ways to signal qualities they like, the more content creators start joining or creating TCC groups and the less visibility is accrued to content without a popular TCC affiliation.

TCCs should be framed around the mission of developing quality standards for user-generated content, organised around general principles such as authenticity, ethics and reliability. Within these mandates, they can also extend further into curation by relevance. Individual TCCs can then concretise these principles for their own small communities, which can range from product reviewers and lifestyle coaches to journalists and politicians. The independence of this infrastructure from platforms allows content creators to use such labels across different digital services simultaneously.

The quality of the content is not to be conflated with its legality, however. In fact, this conflation is often what causes law makers to over-restrict freedom of expression when trying to address challenges such as disinformation. By ‘quality’, this article refers to information that meets verifiable production standards, such as fact-checking, editorial independence, transparency about sources and conflicts of interest, or adherence to other professional norms. Quality standards are pluralistic. Different communities may value different attributes, which enhances the space for freedom of expression of individuals and reduces the pressure on the state to intervene as the guarantor of the quality through state coercion in the marketplace for ideas, through defining what constitutes universally legally restricted content.

Rather than being guilds for entire professions, they would resemble associations of like-minded businesses that produce products of special qualities, such as ‘fair trade’, ‘eco’ or ‘halal’. This time, however, the specific qualities would relate to content production, such as ‘evidence-based’, ‘fact-checked’, ‘appropriate for minors’ or ‘independent’. TCCs can be entitled to better procedural rights, remuneration or other benefits from the platforms or the state.

The article argues that the state must intervene through legislation in three ways:

1. envisaging TCCs as certification groups of like-minded creators that join forces to signal that their members observe self-imposed quality standards;
2. legislating mandates that force platforms to design a communication channel between users and content creators, such as third-party labelling, that would allow users to vote for TCCs by internalising signals of quality of content in their user experience and thus adjusting what they see; and
3. legislating incentives and disincentives for groups of TCCs by means of conditional privileges granted to such groups; those incentives could take the form of enhanced procedural protections, remuneration or negotiation mechanisms, and should be available only to those who have a demonstrable track record of upholding two key requirements: engaging in best efforts to avoid distributing any legally restricted content and active use of internal quality mechanisms to control compliance with self-imposed quality standards.

Once the institutions mature, the law could further proceed by potentially extending TCCs’ role to other tasks that platforms currently centralise, such as adjudicating certain types of legally restricted content or labelling content of non-members.

## 2. Evolution of the Regulatory Landscape

In the late 1990s and early 2000s, the United States, the EU and other jurisdictions adopted clarifications of liability that assured that intermediaries would not be treated as traditional editors who must assume editorial responsibility over their content. This far-sighted policy decision of the first generation, which was championed on the grounds of freedom of expression and innovation, enabled a vibrant ecosystem of user-generated content.^40^ In the following years, the legislators largely adopted a wait-and-see approach before starting to regulate emergent companies.

### A. Early Regulatory Framing of New Media

The first generation of Internet rules was clearly driven by the identical rationale—to encourage investment by giving more legal certainty. As a result, the legal system did ‘steer a path between accusations of censorship and exposure to liability’.^41^ As technology companies started adding more features of content curation—Google search (1998), Facebook’s Top News newsfeed (2009) and YouTube’s Autoplay (2015)—the policy makers started grappling with finding the right frame through which to think about these information products. Increasing platforms’ role of content moderation and curation has led experts to question whether new quasi-editorial activities should be analogised to the editorial responsibility of mass media.

One of the earliest policy frameworks to consider the issue comprehensively was the Council of Europe’s 2011 Recommendation on the New Notion of Media,^42^ which has influenced the case law of the European Court of Human Rights (ECtHR).^43^ The Recommendation proposed thinking of intermediaries who ‘assume an active role in mass communication editorial processes’ as new types of media that must be subject to ‘a graduated and differentiated response’.^44^ This was later developed by the 2018 Council of Europe Recommendation on the Internet intermediaries,^45^ by linking ‘a curatorial or editorial role, including through the use of algorithms’, to ‘a graduated and differentiated response’.^46^

In the post-Brexit-vote atmosphere of the late 2010s, the UK and the EU started to part ways on how they approached the regulation of digital services. In 2018, the European Commission published its Recommendation on Measures to Effectively Tackle Illegal Content,^47^ which subsequently became the blueprint for its 2020 proposal of the DSA. A year later, the UK government published its *Online Harms* White Paper, which became the blueprint for the 2022 Bill leading to the OSA.^48^

The DSA, adopted in 2022, and the OSA, adopted in 2023, both represent the second generation of rules for the Internet.^49^ They accept the framing of graduated and differentiated ‘duties and responsibilities’^50^ by fleshing out specific regulatory expectations that these obligations should entail. Their common denominator is the idea that while intermediaries can be expected to deal with individual items of legally restricted content, especially if they store it, the novel regulatory expectations relate to the design of their systems and processes of content moderation and curation, and user empowerment.

In other words, the key focus of the second generation of rules is on platforms’ accountability for their own systems and processes, not liability for individual pieces of users’ content or behaviour.^51^ The laws shift away from the unproductive debate about liability for third-party content as the only policy lever to achieve change. Instead, they move the conversation to accountability for how systems enabling risks are being designed. The Council of Europe has recently further developed this framework in its Recommendation on Online Safety and User Empowerment.^52^

### B. The Risk of Techno-legal Solutionism

The evolution of ‘graduated and differentiated responses’ as a means to address the growing curatorial powers of intermediaries—while preserving decentralised content production—has generally been welcomed as positive. Yet, some scholars and policy makers caution that the EU and UK legislatures have become overly captivated by a framing that treats the problem as one solvable primarily, or even exclusively, through better technological design. This ‘techno-legal solutionist’ logic risks obscuring the need for broader interventions, including those that address root causes situated in the physical and social world.^53^

There is no doubt that technological design—of interfaces, systems and algorithms—has played a crucial role in creating today’s challenges. McLuhan and Lessig already recognised the regulatory power of design in the 1960s and 1990s, respectively.^54^ More recently, DiResta has extensively documented how algorithmic design shapes the behaviour of both content creators and users, who together co-produce the information ecosystem of digital platforms.^55^ In the political arena, Frances Haugen, a former Meta employee, brought this insight forcefully to global attention by leaking internal documents revealing how Meta’s design choices affected users, particularly children.^56^

However, there is a clear danger in placing excessive faith in new legal mandates for ‘better’ design. As Benkler reminds us: ‘Technology creates feasibility spaces for social practice. Some things become easier and cheaper, others harder and more expensive to do or to prevent under different technological conditions.’^57^ Yet feasibility does not imply causality. Technology may enable or constrain, but it rarely determines outcomes.

The DSA and the OSA are not, in themselves, products of techno-legal solutionism. Nonetheless, their implementation risks succumbing to it. This occurs when regulators expect design interventions to deliver rapid fixes to complex social problems; when they rely on rigid, top-down micromanagement of service design rather than flexible, co-regulatory approaches; and when they overlook the potential of users themselves to act as agents of change, both individually and collectively.

This is why the upcoming Council of Europe Recommendation on Online Safety and User Empowerment puts so much emphasis on user empowerment, which acts as a counterbalance to the traditional top-down measures of online safety. In paragraph 67, the Recommendation puts user empowerment at the centre of the new regime:^58^

The management of online risks should always explore how these risks can be tackled—either wholly or as part of a comprehensive intervention—by granting users more agency over their online experience. User controls of organisation, curation and moderation of content and behaviour, such as adjusting the scope of lawful content that can be prioritised or restricted by specific users, including with the support of third parties of their choice, should be encouraged, and, where appropriate, required. Such user-centred content organisation, curation, and moderation should devolve power but not responsibility over online risks to users.

It is this final dimension to which this article speaks. While the second generation of Internet laws rightly shifts attention from liability for individual items of content to the design of services, regulators and legislators must resist the temptation to become designers of those services themselves. Instead, they should focus on empowering users to deliberate about content quality and to build platform-independent institutions capable of fostering collective sense making and consensus formation online.

For instance, instead of just prescribing all the technical details that platforms used by minors should build, they should equally explore how minors might better adjust the design of services, and co-ordinate with other minors, to better cope with risks; instead of focusing on how platforms should find objective measures to rank the quality of all content, and potentially hide content of lesser quality, the focus should be on giving users tools to prioritise what they value as high-quality content, and to seek better rewards for such content.

Neglecting this dimension, as other scholars have warned,^59^ would mean that the state is bound to fail to properly solve the underlying problems. It would fail by putting too much faith into magical techno-solutionist fixes instead of investing that faith into humanity itself.

### C. Why (New) Institutions Matter

As institutional economists such as Douglass North,^60^ Daron Acemoglu and James Robinson^61^ have shown, societies achieve their greatest advances not through isolated technological breakthroughs, but through the reinvention of institutions that channel human co-operation and trust. From constitutional democracies to welfare systems and international human rights frameworks, history suggests that progress stems largely from institutional innovation.

As noted by Ostrom in her Nobel Prize lecture, ‘a core goal of public policy should be to *facilitate* the development of institutions that bring out the best in humans’.^62^ However, the development of institutions cannot mean centrally planning all their facets. Institutions are organic because, as explained by Fukuyama, they grow out of norms, social behaviour and historical pressures.^63^ The policy interventions can only help to bring to life some outcomes. They can facilitate or incentivise them, but then can only hope that a spontaneous order of some kind will emerge thanks to those interventions. In this spirit, the institutions that I envisage are only designed to facilitate the content creators and their users to develop new quality standards for user-generated content that is trustworthy.

TCCs, the proposed institutions, are not trying to invent something that would not already exist in some shape or form. Deliberation about quality standards is by no means absent from today’s networked public sphere. It does, however, arguably constitute only a tiny minority of the overall communications. Murray has coined the term ‘network communitarianism’ to describe the tendencies of the networked public to supplement the mandated rules with their own bottom-up-developed rules.^64^ Deliberation on Wikipedia pages, Reddit groups and numerous open-source projects shows that quality standards for content production can be developed organically and that members of the community can reach consensus even on very difficult topics.

My point is that such network communities only flourish where the design of platforms allows or facilitates this—and such spaces are the exception to the rule in today’s platform-dominated information ecosystem. In this situation, the goal should be to facilitate the emergence of an institutional design that exceeds the boundaries of individual platforms, and which imposes a meta-structure that facilitates such network communitarianism—the activity of deliberation and seeking consensus, regardless of functionalities and features of a specific platform.

While such institutions might be missing in the DSA and OSA as regulatory frameworks, they are not antithetical to them. On the contrary, if understood as tools for achieving a better information ecosystem, they can be largely seen as complementary to the regulatory goals and mechanisms of the DSA and OSA. In some instances, new institutions can be supported through the enforcement of these frameworks and related non-binding instruments, such as codes of conduct.

## 3. The OSA, the DSA and the Missing Link

The second generation of Internet rules do not radically break from the past. Instead of revolution, they represent an evolution of the early regulatory framing. The new legislative flagships, the OSA and the DSA, left intact key liability exemptions that enable the medium to flourish, and only constructed a new set of regulatory expectations on top of them.^65^

### A. Regulatory Similarities and Differences

The underlying regulatory idea for the OSA and DSA is to diffuse the power of platforms to make centralised decisions about content and users, and overall design of their services. The power is diffused by redistributing it back to individuals and the state, which accepts the task through a regulator and the courts. Under the OSA, the regulator is Ofcom, and under the DSA, the regulator is the European Commission, along with national Digital Services Coordinators.

While the UK and EU legislatures frame their laws slightly differently, and their regulatory approaches differ too, their ultimate target is the same. The goal is to increase accountability of platforms for how they design and operate their services. More specifically, they aim to improve the enforcement of legally restricted content online and empower users to better control their user experience. The laws create due process guarantees, mandate transparency of private decision making and institutionalise constant risk management by a subset of online platforms. These regulatory systems improve the status quo, especially because they coerce companies to much-needed empowerment of individuals.

The OSA uses the concept of ‘user-to-user’ services, which roughly corresponds to the DSA’s notion of ‘hosting’ services, of which ‘online platforms’ are a subset.^66^ Both laws also regulate ‘search engines’.^67^ Both cast their regulatory net widely. While the DSA is a more comprehensive law of the two, the OSA is more far-reaching in its demands from smaller companies.^68^ The DSA and OSA have their own categories of biggest services—Very Large Online Platforms (VLOPs), Very Large Search Engines (VLOSEs), and Category 1 (VLOP-like services) and Category 2A services (VLOSE-type services).^69^

The three key pillars are: regulating content moderation; design and risk management; and transparency. As noted earlier, the DSA and OSA were largely inspired by the popular demands to rein in the design and content moderation processes. Thus, the focus of the first years has been to improve the design of various features of these services, ranging from recommender systems^70^ to interfaces, especially for minors,^71^ and third-party seller verification processes.^72^ Another focus has been on increasing the accountability of their operations through transparency reporting and the facilitation of data access.^73^

Despite similarities, the OSA and DSA differ a lot in terms of their regulatory design. The OSA is mostly construed as a regulator-to-platform relationship, with a minor participatory role for stakeholders. The DSA is construed more as a stakeholders-to-platform relationship, but with a stronger regulator-to-platform dynamic for VLOPs/VLOSEs. This is because for non-VLOPs, self-executable rights of users and their right of private action are realistically the main disciplining drivers for compliance.

Under the OSA, content moderation mistakes are viewed as a content moderation quality problem. Under the DSA, individuals are given directly enforceable rights that they can invoke against companies, with specific expectations of procedural fairness. Where the OSA only envisages super-complaints to regulators, which soon might be complemented by access to data, the DSA creates a complex toolkit of mechanisms put at the disposal of individuals, such as statements of reasons, internal and external appeals, fast lanes for notifications and appeals, access to data and the ability to privately enforce these rights, including by collective redress.^74^

Besides super-complaints and an *ad hoc* role for auditors, the OSA does not generally consider any institutions other than Ofcom. The DSA, in contrast, puts less emphasis on the regulator with respect to content moderation and relies on a complex ecosystem of non-state actors to work. The DSA foresees a role for: trusted flaggers of illegal content;^75^ user groups who are given rights to defend content creators;^76^ researchers who study risks and mitigation measures posed by platforms;^77^ auditors to review the risk assessments;^78^ and out-of-court dispute settlement bodies^79^ that hear individual content moderation grievances of users. These non-state actors under the DSA are key to diffusing the platform accountability into an exercise of many actors, thus avoiding the concentration of powers with the state. However, their role is one of watchers who perform certain tasks of the regulatory oversight, but do not try to increase the deliberation of users and content creators, or promote consensus among them.

Trusted flaggers, user groups and out-of-court dispute settlement bodies help to augment the ability to fight back by individuals who are victims of user-generated content or its moderation. Researchers and auditors augment the public’s ability to study the risk and mitigation measures posed by design, functioning and use of the digital services. They all help to professionalise the process of content moderation and risk management—but only along the content moderation and curation lines drawn by the platforms and the state.^80^ The states and platforms thus remain the dominant powers in formulating what types of content are allowed on the platforms and what gets to be shown to their users.

### B. Power to Set the Rules for Content

During the legislative process, the power to formulate content moderation rules, as opposed to how to apply them, has been thematised only marginally. Very few seriously questioned the autonomy of platforms to set their own rules in addition to content rules foreseen by a legal system. This is understandable. Even the latest case law of the ECtHR confirms that platforms have a freedom of expression right to not carry content they do not want to carry, and that the state can only coerce them to carry it if there is a ‘pressing social need’.^81^ Thus, whether presented as a contractual freedom^82^ or a right to remain silent, platforms’ choice of contractual content moderation rules is a starting point.

The only context in which this right of platforms was questioned concerned legacy media. Newspapers and broadcasters argued that their editorial content should not be subject to additional assessment and rules imposed by platforms. Thus, effectively, the legacy media sought an exemption from the content moderation of their lawful content by platforms, as they obviously must observe states’ rules on what constitutes legally restricted content. The efforts to do so succeeded in some form in the OSA.^83^ While they initially failed to materialise under the DSA, soon afterwards, the EU legislature adopted them in some form in the European Media Freedom Act (EMFA), which complements the DSA.^84^

Both section 18 OSA and article 18 EMFA now constrain the power of platforms, but only procedurally. The starting point remains the right of platforms to design their terms and conditions as they see fit, although observing the interests of others in some form.^85^ Neither provision amounts to an exemption from platforms’ content moderation rules.

In the UK, a ‘recognised news publisher’ who disseminates content via so-called Category 1 services enjoys increased protection of their journalistic content. Recognised news publishers, according to section 56 OSA, must: principally publish news-related material as their business; produce it by different persons under editorial control; observe the editorial standards of a standards code; have policies and procedures for handling and resolving complaints; and be properly based in the UK. Unless the dissemination of publishers’ content can lead to legal liability for the platform, an advance notice to publishers is necessary before removing, restricting or labelling it for the users.^86^

In the EU, ‘media service providers’ must first undergo an onboarding process with each VLOP in order to, *inter alia*, demonstrate editorial independence, governance by at least a self-regulatory mechanism governing its editorial standards and commitment not to use AI-generated content without editorial review.^87^ Once onboarded, such media service providers are owed an advance notice, a *de facto* obligation to carry content for 24 hours and priority appeals.^88^ However, such protections do not apply if content moderation relates to illegal content or the protection of minors, or is necessary to comply with the DSA’s risk management system, which effectively entirely weakens the obligations.^89^

Directionally, section 18 OSA and article 18 DSA are understandable. Both provisions rightly identify that we are currently missing preferential treatment for reliable and authentic content in the sea of user-generated content, which is increasingly also AI-generated.^90^ After all, legacy media, despite their imperfections, represent one of the non-state institutions that humanity has developed to produce reliable information through observance of editorial standards in the newsroom.

That being said, both interventions are also unlikely to succeed. Neither is likely to achieve much change in the information ecosystem. Firstly, the ecosystem is dominated by user-generated content that does not come from news publishers.^91^ Thus, any preferential treatment will have a minimal effect. Secondly, the protections granted are weak for a reason. News publishers as a business class are hardly a homogeneous group that can be presented as synonymous with high editorial standards. The *Daily Mail*, for instance, would qualify as a ‘recognised news publisher’ under the OSA, even though Wikipedia’s editors have long treated it as an unreliable source that cannot be used as evidence for factual claims.^92^

These two reasons also point to what is missing in these acts. The regulations are failing to create a comprehensive communication channel for the quality of content. The laws do not help users to tell high-quality, reliable content apart from that which is not. Clearly, in the networked public sphere, such differentiation cannot be based on pre-Internet standards of quality. Such standards cannot be limited to one profession or a country, but must be universally open to everyone—people of different walks of life who disseminate information online while engaging in activities ranging from commentary, news reporting, product reviews or lifestyle coaching.

### C. Mostly Defensive Empowerment Obligations

The DSA and OSA do not envisage any explicit constructive empowerment obligations, that is, obligations giving users an option to influence what types of content they *want* to see. Most of the focus for both laws is on what users should avoid seeing or experiencing. This defensive empowerment dominates because it maps well to the original goal of these laws to improve the enforcement of legally restricted content online.

The OSA, in contrast to the DSA, is more explicit in envisaging empowerment duties in sections 14 and 15. Such duties only apply to Category 1 services, that is, the biggest platform-like services in the UK. However, the implicit focus is on the kind of content that the user does not want to see. Section 15(3) OSA speaks of features to effectively reduce the likelihood of the user encountering some content or alert the user to some kind of content. Section 15(9) OSA envisages control to filter out non-verified users, either by preventing such users from engaging with content creators or by giving users the option to reduce the encounter of content of non-verified users.^93^ Sections 14 and 16 confine ‘alerting to content’ to only ‘defensive vision’ of the empowerment when they speak about categories of content that relates to suicide, eating disorders, self-injury, discriminatory abusive behaviour or hate.^94^

Under the DSA, EU users are guaranteed an option to switch off VLOPs’ personalised recommender systems.^95^ In addition, empowerment efforts under the risk management system, whether under article 28 (minors) or article 35 (general), are focused on giving users options to avoid seeing or encountering something. For instance, for minors, platforms are expected to offer digital services that have many features turned off, such as recommendations of other accounts, filters that can be associated with negative effects on body image, self-esteem and mental health, or features that may contribute to excessive use, such as the number of ‘likes’, ‘streaks’ or autoplay.^96^ For elections, VLOPs are expected to ‘establish measures to reduce the prominence’ of deceptive and misleading content generated by AI, and to reduce the prominence of disinformation by engaging with fact-checkers, which arguably includes user empowerment, not only top-down design decisions of platforms.^97^

There are, however, subtle ways in which the DSA hints at the possible increase of power of users to select information they want to see, and thus directionally support the idea of constructive empowerment. Guidance on article 28, for instance, states that platforms should ‘Prioritise “explicit user-provided signals” to determine the content displayed and recommended to minors’, as opposed to ‘implicit engagement-based signals’, that is, signals inferring user preferences from their activities on a platform, such as time spent viewing content and click-through rates.^98^ Similarly, guidance on article 35 in the context of elections states that VLOPs must ensure ‘that recommender systems are designed and adjusted in a way that gives users meaningful choices and controls over their feeds, with due regard to media diversity and pluralism’.^99^

Content labelling, whether by content creators themselves or third parties, is an explicit signal. If users were to gain the ability to prioritise what they see based on such signals, the intervention would increase their control over what they see. At the moment, labelling is mostly discussed in the context of fact-checking, which is arranged by platforms that pay third-party fact-checking organisations as part of their risk mitigation strategies.

### D. Unlike Vertical and Horizontal Interoperability

Before proceeding to the detailed discussion of the proposal itself, it is important to highlight what the proposal is not about. Constructive empowerment increases the control of users to see more content that they want to see. Such empowerment can also be achieved through two other policy proposals that are currently being discussed: (i) the decoupling of content curation through middleware providers; and (ii) the horizontal interoperability of digital services.

An increasing number of scholars are proposing the decoupling of the activity of content curation from the core platform business through third-party players known as middleware providers.^100^ The integration of such players in the digital services is a form of vertical interoperability. The basic idea is grounded in the concerns about the increased concentration of platform market power, which should be counterbalanced by the decoupling of content curation functions from the function of storing information and connecting users.

In this conception, the regulation should create a space for new providers known as middleware providers, who could offer recommendations curated according to alternative ranking, labelling or content-moderation rules to users who decide to use them. This way, the most powerful market players will be exposed to more competition *within* the ecosystem of their own services when it comes to what users see. Users would be allowed to vote with their feet and select alternatives, which, it is hope, will discipline the standard offering of curated content. The Council of Europe’s Recommendation on Online Safety and User Empowerment, for instance, recommends that states explore this option.^101^

An alternative proposal is to discipline the most powerful market players by increasing competition for the core digital service that connects users, such as social media, by mandating that they interoperate with those of their competitors.^102^ The benefits of horizontal interoperability are much more difficult to achieve because some competitors must first build an alternative digital service, such as social media, keep maintaining it and attract users to use it instead. The hope is that users will recognise the value of competing offers and switch more easily because they can continue to communicate with their contacts on the platform they have left.

While both of the above conceptions have their merit and are being explored on different policy levels,^103^ they are also grounded in different justifications than the proposal presented here. Vertical and horizontal interoperability are guided by concerns of market power concentration, which by definition means that they would be limited to the biggest players on the market. The focus on building complementary institutions presented in this article has a much broader application. Its justification is grounded in the indispensability of deliberative institutions, which are essential for any democracy and marketplace of ideas.^104^

Nevertheless, the concept of trusted content creators requires some regulatory mandates to support constructive empowerment of users and some degree of vertical interoperability to facilitate the communication between users and content creators. However, in contrast to the proposals for unbundling, or horizontal interoperability, the proposal requires much smaller interventions in the design, is not limited to the biggest market players and is not preconditioned on the existence of other platform market players to work. It is, however, reliant on the creation of a vibrant ecosystem of self-organised content creators and active users.

The core idea is to develop portable institutions that are independent of platforms. Because it operates on a cross-platform basis, organised around content creators, it can be incorporated into digital services that are used for public as well as private communication. Once they exist and can be communicated to users, the labels, and thus the choice, follow content creators everywhere they choose to be followed, including to new market entrants.

## 4. Trusted Content Creators as the Missing Link

Independent certifying groups struggle to emerge because neither states nor platforms create incentives for them, and because collective-action problems—limited resources, high co-ordination costs and global fragmentation—make organisation difficult. Moreover, platform design offers no reliable way for external quality signals to influence user experience, leaving groups with no reason to invest in certification they cannot effectively communicate or monetise.

### A. Labels as Constructive User Empowerment

The first step is to create a communication channel between users and third parties that can help them to curate the content. The goal of such a communication channel is to allow users to express their preferences for content in a more comprehensive way than through regular engagement features for individual creators or items of content, such as likes or follows. A well-functioning communication channel is a precondition because any system where consumers are to take the centre stage by voting with their feet needs to first grant them a way to directly listen and speak to producers. Only if they can listen unobstructedly can they properly reward or punish producers who address them.

A great example of an infrastructure creating such a communication channel is Bluesky’s third-party labelling system.^105^ It allows third parties to attach labels to any content on its platform. Users who subscribe to such labellers are, in turn, able to internalise them in their user experience. For instance, if a third party labels random content as ‘offensive’, ‘not safe for work’, ‘containing spoilers’ or ‘evidence-based’, the user can decide to hide, prioritise or blur all the content corresponding to that label. This way, the user’s preference is superimposed on the regular user experience on the services, including on the results of its centralised recommender systems. The labelling allows users to vote for what they want and do not want to see.

This labelling infrastructure offers many benefits. For third-party labellers, it creates a new communication channel concerning the user-generated content on the platform, which can be used to communicate signals related to the content’s overall quality or individual qualities. It facilitates mass voting by users on user-generated content in a much more comprehensive way than simple micro-signals of user engagement. The communication channel is also not fully controlled by platforms. It allows the institutions to bypass the algorithmic decision-making power of the platforms, provided that such third-party labellers can convince the users to implement their labels in the settings.

There are historical parallels for such changes. Prior to the mass production of goods, trust was primarily personal and local. Buyers trusted their local merchants to sell them quality products. The merchant rather than the product was the primary bearer of trust.^106^ As explained by Strasser, when standardised products began to circulate through impersonal and long-distance markets, brands of producers became the new technology of trust; they allowed firms to transfer the trust signals from the local shopkeeper to the product package.^107^

With brands as a new communication channel between consumers and producers, the importance of the curatorial role of local merchants became less important. The over-the-counter model soon shifted to a self-service model at ever-larger supermarkets, in which retailers play a very limited curatorial role. The supermarket effectively transferred responsibility for choice from the merchant to the shopper.^108^ By doing so, it made branding and packaging essential tools for attracting attention and conveying trust.

Thus, being able to communicate labels is not a trivial change to the ecosystem. It creates a space for a new layer of content curation and thus of developing a trust relationship with consumers. While content creators can build their individual brands for their information products already today, they are strongly dependent on the over-the-counter model of content curation provided by platforms, in which the algorithm is the key factor of their success.

Trademarks allow producers to signal the source and consistent quality of their goods or services, incentivising them to invest in maintaining that quality over time. As Posner notes, trademarks ensure that ‘the producer who has won consumer approval for his product through investment in quality and advertising is not deprived of the benefits of that investment’.^109^ Certification marks work similarly for groups: they reward collective investment in quality by protecting the economic and reputational benefits of the group’s shared standards, so long as members’ quality is effectively supervised.

Third-party labelling has recently been envisaged by the Council of Europe in its Recommendation on Online Safety and User Empowerment, which states the following:^110^

Platform design should enhance the ability of users to make informed choices about the content they engage with, including by facilitating third-party labelling of lawful content by experts, fact-checkers, or communities. Users should be allowed to act upon such labels in their settings to further personalise their online experience by hiding or prioritising content corresponding to specific labels.

The third-party labelling has the potential to create many communities of experts who curate someone else’s content for their user communities. Its scope of application is thus much broader. For the purposes of this article, however, its role as a communication channel *about* qualities of user-generated content is critical for the key deliberative and consensus-building institution—TCCs.

### B. Trusted Content Creators

In the networked public sphere, the quality control is dispersed among various nodes that organise the world’s information. As explained earlier, we are missing a non-state institution that could inject consensus-based quality signals into the ecosystem of content creators. Without it, content creators who observe quality standards in their content production have a hard time signalling quality credibly to others. If their signals were legible and actionable by users, groups of like-minded content creators could collectively be more easily rewarded by users’ trust.

Many legal systems, including those of the EU, the UK and the United States, have known so-called certification trademarks for years. Certification trademarks allow organisations to gain exclusive rights to signs that signify, among other things, the ‘mode of … performance of services, quality, accuracy or other characteristics … from goods and services which are not so certified’.^111^ Unlike their well-known cousins, geographical indications, which are tied with local communities in specific geographies, certification marks can be organised entirely around quality standards or characteristics. Individual businesses can use the certification trademarks if they gain authorisation from the owner of those marks, usually an association, which confirms that their goods conform to the mark’s specifications. The certifying authority must also supervise compliance on an ongoing basis.^112^

TCCs would act as such certification entities and would be invested in supervising their members because the reputation for a label is always earned and lost collectively. TCCs would thus be exposed to a mixture of co-operative and competitive dynamics that have been well documented in the area of geographical indications.^113^ Individual content creators would be motivated by potentially obtaining premium attention, reputation and profits.

If TCCs are a proxy for quality, this would channel the attention of users more to reliable content of higher quality. Individual content creators, through TCC groups, pool resources to target consumers who are willing to pay for attributes of content that are not found in the generic content. In exchange, content creators inevitably must delegate some level of quality control to the collective body.

The policy goal behind TCCs as institutions is to create spaces for deliberation on quality standards. The mechanism for the deliberation resides in their collective nature because TCCs rise and fall as a group. The groups start by defining their core values that set them apart from the generic competition. Special qualities captured in the common practice rules can include typical standards concerning editorial independence, fact-checking, transparency about creative processes, finances, conflicts of interest, authenticity of content and rules about how they produce content.

Since violation of common practice rules or laws by a single member can reflect badly or even have legal consequences for everyone in the group, the peers in the group are incentivised to police each other to keep the group well regarded. Thus, a further forum for deliberation is the complaint mechanism, which would hear petitions from members or the public. We can imagine deliberation in such groups as being akin to accountability discussions of professional bodies of professionals, such as lawyers, but also as debates on Wikipedia talk pages where Wikipedians discuss edits, or discussions of open-source developers before they adopt specific changes to code.

Thus, TCCs facilitate deliberation about content standards in two ways: through the formulation and acceptance of the group-specific common practice rules and through the resolution of disputes that arise around individual cases of adherence to such rules. In all these cases, TCCs act as facilitators of group consensus. While they do not represent deliberation among all content creators in the class, the gradual adoption of TCCs by users through labels and major content creators can turn them into *de facto* standards.

The key mechanism for connecting such *deliberation* with *voting* by users works through the system of labelling. TCCs would automatically give exclusive labels to the content of their individual content creators. Those labels, similar to protected geographical indications for physical products, would serve to distinguish premium content from that of lesser quality, with the goal of reaping premium attention and remuneration. Such voting is an important feedback mechanism for TCCs, but also a signal of adoption for other content creators who have not joined the existing TCCs.

For users, TCCs create an extra granular option to decide what to read and watch. They can voice their user preferences via labels that are curated independently of platforms. They do not have to make individual decisions about each content and content creator, but only make a (dynamic) decision about a group of such creators.

### C. Necessary Incentives and Disincentives

The proposed system requires a legislative intervention with a clear mandate. The legislatures must specify how the providers would communicate the existence of TCC groups to users, and what subscribing to their labels would mean for them.

The trickiest of all questions is clarifying the mandate of such groups. The law needs to navigate two dangers: risks of value-free content aggregation unrelated to quality standards and the risk that the state turns certification of TCCs into a regulation of content by the back door, by conflating legality and quality of content. To ensure a meaningful *quid pro quo* for society, the legal framework should set basic parameters within which groups define their common practices. The mandate should be broad enough to accommodate diverse ways of demonstrating ‘quality’ but not so broad as to permit capture. Each TCC would specify what those terms mean for its members and enforce them internally, with legal minimums tied to compliance with existing restrictions on legally restricted content. Breaches of these minimums should trigger serious consequences, such as suspension of the group’s label or similar sanctions.

Once implemented, the system would give creators a choice: remain independent or join a TCC to gain access to a larger audience. Membership requires delegating some quality control to peer experts, which increases legitimacy and resembles accountability within professional bodies. TCCs could also offer practical benefits—assistance with moderation disputes, liability insurance, copyright support, expedited appeals or collective bargaining—while vetting members and expelling them where needed. Internal complaints procedures and peer review would provide independent supervision and build trust through deliberation.

The by-product of such organised peer scrutiny is a new mechanism for signalling the quality of user-generated content. This resembles scientific journals, which act as filtering institutions that enhance trust and visibility without necessarily promoting substantive consensus or a shared agenda. Just as academics may publish outside journals but lose the benefits associated with peer-reviewed outlets, creators could operate independently yet forgo the visibility, trust and attention premiums associated with TCC membership.

To formulate the bargain clearly, the law should keep the mandate for TCCs open but grant enhanced rights only conditionally to those TCC groups that have a demonstrable track record of upholding two key requirements: best efforts to avoid distributing any legally restricted content and active use of internal quality mechanisms to police self-imposed quality standards.^114^ But to avoid regulation of content via the back door, regulators should only certify that TCC groups have procedures and processes for such activities, not whether the self-imposed quality standards are good for society, nor whether members are experts. In this sense, the system must significantly differ from expertise certifications, such as those for out-of-court dispute settlement bodies or trusted flaggers under the DSA. Users, not regulators, should be the judges of quality.

The final design feature of the system would be to address the problem of collective action that is likely to slow down the creation of TCC groups. The collective action problem is posed by ‘disincentives that tend to discourage joint action by individuals in the pursuit of a common goal’.^115^ The key disincentives in this case are likely to be: transaction costs; the need to overcome resistance from platforms; the cost of consensus-building around quality standards; and the cost of enforcing them against its members.

Nothing overcomes the collective action problem better than great economic benefits. The main draw is the promise of increased attention and potentially higher remuneration that comes with recognised usage of quality labels. The law could address remuneration by platforms by stipulating that qualified TCC groups are owed ‘substantially higher remuneration’ than generic content, and leave it to the TCC groups to negotiate with the platforms, subject to some dispute resolution mechanism.

Another approach is to assign some TCCs delegated public roles and compensate them for carrying them out. Through peer-based quality control, TCCs already perform a form of delegated self-regulation that benefits both the public and the platforms by creating credible signals about content quality. The state could provide monetary incentives for TCC formation. Where TCCs take on functions such as third-party labelling or delegated decision making around non-manifest legally restricted content, platforms themselves could be required to contribute financially because TCCs help mitigate externalities created by platform operations.

As noted earlier, the incentives should be *conditional* upon two requirements that would have to be demonstrated: best efforts to avoid distributing any legally restricted content and active use of internal quality mechanisms to police self-imposed quality standards.

Thus, TCC groups that are not certified in this way only benefit from the direct communication channel with users, but nothing else. However, to prevent abuse even by those groups, platforms should be obliged to sanction TCCs whose members repeatedly manifestly violate the law, extending existing DSA mechanisms^116^—such as suspending accounts to entire groups where appropriate. Sanctions could include suspending or blocking the distribution of labels or even disabling accounts. Similarly to the DSA, such sanctions could also be privately enforceable. TCC groups, on the other hand, should benefit from the redress mechanisms.

If TCCs succeed in directing greater user attention to lawful content because users effectively vote for content that is lawful, even if awful for some, the regulators should stand by and, at best, observe and study to make a case for the legislature.^117^ The system is meant to avoid making regulatory shortcuts of deplatforming voices that are lawful but objectionable. If platforms make such decisions voluntarily, the law already includes some protective mechanisms in extreme cases.^118^

**Figure 3.**
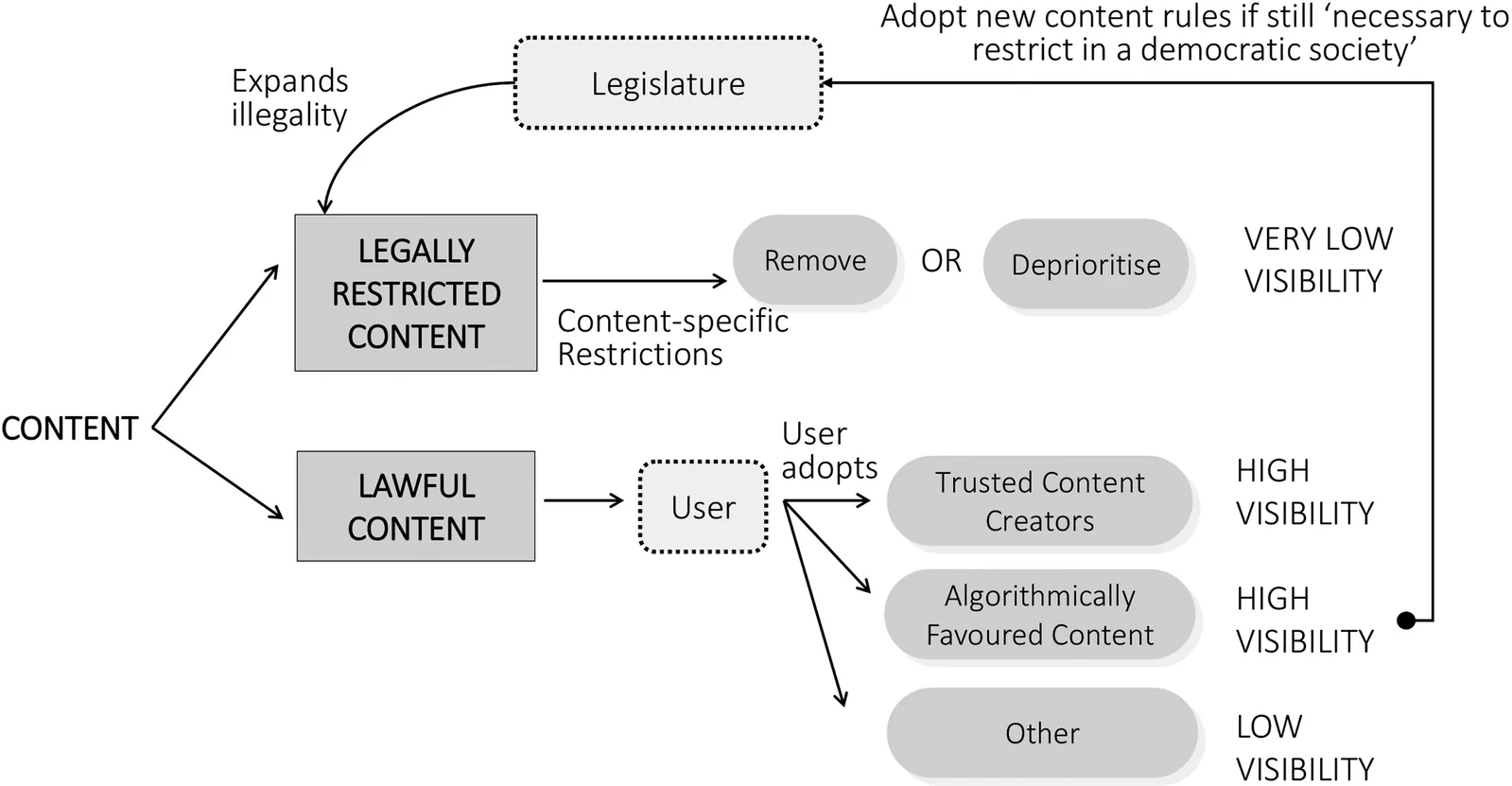
The figure shows that there are three states for lawful content. High visibility for content distributed by platform algorithms and TCC groups, and low visibility for other content. The legislatures, however, can always reclassify any such lawful content as legally restricted to reduce its visibility.

If TCC groups promoting lawful but problematic content are formed and become successful among users, the public needs to make a case to legislators that new content rules for everyone are ‘necessary in a democratic society’. The existence and practice of such TCC groups, but also of other competing TCCs, can then improve the public debate about the necessity of new content rules (see Figure 3).

Some might object that such a solution risks creating a new type of filter bubble and echo chamber.^119^ It is also true that new user-controlled TCC lenses can further fragment user experience, as people have different preferences. However, organic ‘user-controlled’ bubbles are preferable to manufactured ‘market-imposed’ bubbles, and the state has at its disposal much more targeted remedies to address this, such as so-called must-carry mandates requiring media to publish information in the public interest.^120^ Where there is a ‘pressing social need’,^121^ such mandates can impose shared media diets more effectively and are not precluded by the proposed system. TCCs are not meant to be the final solution for how to address content online. That is unrealistic. Human behaviour constantly develops. But TCC can be seen as a deliberative system that gives users a genuine voice and choice over what to read online, and a process to deliberate about what the state ought to do next.

## 5. Concluding Remarks

In his book about mass media and ownership concentration, Edwin Baker argues that the widest possible dispersal of media power reduces the risk of the abuse of communicative power.^122^ He argues that:^123^

The basic standard for democracy would then be a very wide and fair dispersal of power and ubiquitous opportunities to present preferences, views, visions. This is a democratic distribution principle for communicative power—a claim that democracy implies as wide as practical a dispersal of power within public discourse.

The rise of algorithmic curation by platforms fortifies them as the key gatekeepers of information flows. Content creators currently depend on platforms’ algorithmic curatorial systems to their reach audiences. This gives platforms ‘opinion power’^124^ that can be used for political ends. Despite this, the curatorial power of platforms is not subject to any safeguards comparable to those known from media legislation. Even the latest European regulations, such as the EU DSA and UK OSA, do little to disperse the curatorial power of platforms.

TCCs are a solution to the concentration of such communicative powers by platforms.^125^ They decentre, rather than centralise, and disperse those powers by giving users more choice of what they see. This choice could profoundly reshape the information ecosystem, especially if such empowerment is linked with certification groups developing content quality standards. Finding and defining quality standards through interactions with users serves an additional and important function: it creates an alternative to state-led certification of qualities and reduces the need for top-down imposition of substantive standards. It builds upon the legacy of the *Handyside* judgment, where the ECtHR rejected the idea that the state should be deciding what constitutes acceptable or proper expression, invoking the democratic values of pluralism, tolerance and broadmindedness.^126^ The proposed TCC system is not a state intervention to define what content is acceptable or proper. It does not favour any viewpoint in the range of lawful content. Rather, it is a content-agnostic intervention to improve the exchange of views on the marketplace for ideas. Its goal is for people to be better able to choose what they read online. It does not give people the power to choose what *others* should and should not read. In this way, it enhances rather than limits freedom of expression in general. The system will not end legislative debates aimed at revising content rules. But, similarly to social conventions, it reduces the pressure from having the state micromanage our lives.

Josiah Ober famously argued that one original meaning of democracy is not simply the rule of the majority but the collective capacity of groups to do things through knowledge-based co-ordination.^127^ TCCs operate in this vein: they aggregate and codify dispersed knowledge, align the behaviour of their members and let quality norms emerge organically rather than through legislative fiat. This logic resonates with Hayek’s account of markets as mechanisms that collect and evaluate tacit knowledge held by dispersed actors.^128^ TCCs can therefore be seen as extensions of market participants who respond to user-generated ‘market signals’ when users adopt or reject their labels. Voting by users becomes an additional layer of knowledge aggregation that disciplines and revises TCC standards from the bottom up.

The proposed system of TCCs is not limited to non-commercial forms of curation; indeed, the law should recognise that quality standards often develop within profit-maximising settings. Established curators—newspapers, music labels, film studios—or new curators could collectively use TCCs to create new ways to signal the qualities of their offerings. Because the underlying mandate of all TCCs is quality, user choice would become more tightly connected to certified attributes. Groups could also create sub-labels to reflect differing degrees of relevance, provided that all such curation is tied to certified qualities rather than value-free sorting. This approach would benefit a broad range of platforms beyond social media. Marketplaces, search engines, maps, app stores and streaming services could all offer new filtering dimensions based on TCC certifications.

The Internet needs a shift from ‘over-the-counter’ curation by platforms to a system where consumers and content creators can communicate about the qualities of their products directly. In a recent consumer survey study for the European Commission, 40% of users expressed interest in using third-party solutions to ‘filter out illegal or harmful content’ and 26% in solutions ‘to recommend content’.^129^ Similarly, in a study examining people’s preferences in 26 countries from around the world, people consistently expressed a strong preference for being their own choice architects. People across a broad range of political systems, cultural backgrounds and platforms want more control over their online environments than they have at the moment.^130^

Thus, individuals are clearly aware of the choices that are missing due to too much focus on corporate and governmental interventions. The proposed system of TCCs develops the legal infrastructure needed so that society can develop its own ‘professional filters’. Such institutions, to paraphrase Ostrom, ‘can bring out the best in humans’ because they improve not only consumer choice, but also deliberation.^131^ Current regulations, such as the OSA and DSA, focus primarily on compelling platforms to better enforce existing laws, overlooking the parallel need to empower users to deliberate and define appropriate content quality standards. Without a trusted ‘third option’ that allows individuals to opt into self-chosen standards, debates over content governance default to binary demands for centralised state or platform-wide content restrictions.

TCC labels implemented by users do not remove or otherwise block low-quality content. However, through their wide adoption by users, labels can make low-quality content either invisible or less visible, but only to people who opt to use the labels. The policy intervention thus protects freedom of expression and is superior to state- or platform-imposed policies to suppress such content centrally. It allows people to better navigate between many different quality standards of lawful content and allows societies to continue to deliberate about standards for user-generated content on a global or regional scale, without reinstating centralised editors.

If flooding the zone with false or low-quality content reliably captures attention—and if producing falsehoods is cheaper than debunking them—the solution is not to rank all information, but to create credible signals for content of higher quality. Credible premium labels can accelerate that process by giving users new lenses through which to decide what to read online. As these signals become widespread, the constant presence of generic low-quality information will matter less because it will be less visible. Someone will always be wrong on the Internet;^132^ the point is not to reward them.

## Footnotes

1 Jürgen Habermas, *A New Structural Transformation of the Public Sphere and Deliberative Politics* (Polity 2023) 13, 15, 20 and 31.

2 Edwin C Baker, *Media Concentration and Democracy: Why Ownership Matters* (CUP 2006) 7.

3 Habermas, *A New Structural Transformation* (n 1) 59.

4 Jürgen Habermas, *The Structural Transformation of the Public Sphere* (Polity 1989) 175.

5 Yochai Benkler, *The Wealth of Networks: How Social Production Transforms Markets and Freedom* (Yale UP 2007) 370–1.

6 See Tim Wu, *The Attention Merchants: The Epic Scramble to Get Inside Our Heads* (Knopf 2016); Thomas H Davenport and John C Beck, *Attention Economy: Understanding the New Currency of Business* (Harvard Business Review Press 2002).

7 As explained by Benkler (n 5) 4, communications via telegraph, radio, TV and print on a large scale required a lot of physical capital, hence information and cultural production had to take the shape of a market industrial model.

8 Habermas, *A New Structural Transformation* (n 1).

9 Benkler (n 5) 10.

10 Renée DiResta and Rachel Kleinfeld, ‘For Expertise to Matter, Nonpartisan Institutions Need New Communications Strategies’ (Carnegie Endowment, 2025) <https://carnegieendowment.org/research/2025/09/communications-social-media-nonprofit-institutions-new-media-environment?lang=en 3> accessed 25 November 2025.

11 ibid 3.

12 Habermas, *A New Structural Transformation* (n 1) 49.

13 Lawrence Lessig, *Code and Other Laws of Cyberspace* (Basic Books 1999); Nick Couldry, *The Space of the World: Can Human Solidarity Survive Social Media and What If It Can’t?* (Polity Press 2024); Linda Mulcahy, ‘Architects of Justice: The Politics of Courtroom Design’ (2007) 16(3) Social & Legal Studies 383 (on impact of architecture of court houses).

14 Habermas, *A New Structural Transformation* (n 1) 55 (‘yet professionally unfiltered’).

15 ibid 13, 15, 31 (importance for democracy), 35–7, 55 (professional filters) and 53 (qualities).

16 Natali Helberger, ‘The Political Power of Platforms: How Current Attempts to Regulate Misinformation Amplify Opinion Power’ (2020) 8 Digital Journalism 846.

17 Daron Acemoğlu and others, *The Economic Imperative of Investing in Public Interest Media* (Forum Information Democracy 2025) <https://informationdemocracy.org/wp-content/uploads/2025/09/The-Economic-Imperative-of-Investing-in-Public-Interest-Media.pdf 7> accessed 25 November 2025.

18 ibid 8.

19 Joseph Stiglitz, Anya Schiffrin and Dylan Groves, ‘Journalism for Development: The Role of Journalism Promoting Democracy and Political Accountability and Sustainable Development’ (UNESCO 2024) 8; Acemoğlu and others (n 17) 6, 9; Christian Bjørnskov, ‘Social Trust and Economic Growth’ in Eric M Uslaner (ed), *The Oxford Handbook of Social and Political Trust* (OUP 2018).

20 See Philipp Lorenz-Spreen and others, ‘How Behavioural Sciences Can Promote Truth, Autonomy and Democratic Discourse Online’ (2020) 4(11) Nature Human Behaviour 1102; Tom Cunningham, ‘Ranking by Engagement’ (blog, 28 April 2023) <https://tecunningham.github.io/posts/2023-04-28-ranking-by-engagement.html> accessed 25 November 2025.

21
*Federal Trade Commission v Meta Platforms Inc, Revised Memorandum Opinion* (No 1:20-cv-03590-JEB, US District Court for the District of Columbia, Judge James E Boasberg, 2 December 2025) Document 705, 8.

22 Couldry (n 13) 81 and 91.

23 James Hamilton, *All the News That’s Fit to Sell: How the Market Transforms Information into News* (Princeton UP 2004) 38ff.

24 Matthew Gentzkow and Jesse M Shapiro, ‘What Drives Media Slant? Evidence from U.S. Daily Newspapers’ (2010) 78(1) Econometrica 35; William B Reddaway, ‘The Economics of Newspapers’ (1963) 73(290) The Economic Journal 201; Matthew Gentzkow, Jesse M Shapiro and Michael Sinkinson, ‘Competition and Ideological Diversity: Historical Evidence from US Newspapers’ (2014) 104(10) American Economic Review 3073.

25 Matthew Gentzkow and Jesse M Shapiro, ‘Competition and Truth in the Market for News’ (2008) 22(2) Journal of Economic Perspectives 133.

26 Hunt Allcott and Matthew Gentzkow, ‘Social Media and Fake News in the 2016 Election’ (2017) 31(2) Journal of Economic Perspectives 211, 218–19; Vian Bakir and Andrew McStay, ‘Fake News and the Economy of Emotions: Problems, Causes, Solutions’ (2018) 6(2) Digital Journalism 154.

27 George A Akerlof, ‘The Market for “Lemons”: Quality Uncertainty and the Market Mechanism’ (1970) 84(3) Quarterly Journal of Economics 488.

28 See Hamilton (n 23); Michael R Darby and Edi Karni, ‘Free Competition and the Optimal Amount of Fraud’ (1973) 16 Journal of Law and Economics 67.

29 See Deepak Nayal, ‘Why Is Interest Graph More Interesting than Social Graph?’ (*Medium* 2025) <https://medium.com/@dnayal/why-is-interest-graph-more-interesting-than-social-graph-59d5e105d567> accessed 25 November 2025.

30 See Reuters, ‘Meta’s Facebook Revamping Main Feed to Attract Younger Users’ (2022) <www.reuters.com/technology/metas-facebook-revamping-main-feed-attract-younger-users-2022-07-21/> accessed 25 November 2025.

31 New America, ‘Case Study: Amazon’ <www.newamerica.org/oti/reports/why-am-i-seeing-this/case-study-amazon/> accessed 25 November 2025.

32 See Regulation (EU) 2022/1925 of the European Parliament and of the Council of 14 September 2022 on contestable and fair markets in the digital sector and amending Directives (EU) 2019/1937 and (EU) 2020/1828 (Digital Markets Act) [2022] OJ L265/1, art 3 (important gateway); Digital Markets, Competition and Consumers Act 2024, s 6 (strategic significance).

33 Philip Napoli, *Social Media and the Public Interest: Media Regulation in the Disinformation Age* (Columbia UP 2019); Rasmus Kleis Nielsen and Sarah Anne Ganter, ‘Dealing with Digital Intermediaries: A Case Study of the Relations Between Publishers and Platforms’ (2018) 20(4) New Media & Society 1600.

34 Many NGOs see such proposals only as one of many interventions. See Knight Georgetown Institute, ‘Better Feeds: Algorithms That Put People First’ (2025) <https://kgi.georgetown.edu/research-and-commentary/better-feeds/> accessed 25 November 2025; Toda Peace Institute, ‘Prosocial Tech Design Guidance’ (2025) <https://toda.org/assets/files/research/blueprint-on-prosocial-tech-design-governance.pdf> accessed 25 November 2025; Panoptykon Foundation, ‘Prototyping User Empowerment’ (2023) <https://panoptykon.org/sites/default/files/2023-11/peoplevsbigtech_panoptykon_prototyping-empowerment_brief_20112023.pdf> accessed 25 November 2025; Panoptykon Foundation, ‘Safe by Default’ (2024) <https://panoptykon.org/sites/default/files/2024-03/panoptykon_peoplevsbigtech_safe-by-default_briefing_03032024.pdf> accessed 25 November 2025; Glen Weyl and others, ‘Prosocial Media’ (2025) <https://arxiv.org/abs/2502.10834> accessed 25 November 2025; Napoli (n 33).

35 For similar critique from the perspective of increasing platforms’ opinion power, see Helberger (n 16) 848.

36 Friedrich Hayek, ‘The Use of Knowledge in Society’ (1945) 35(4) American Economic Review 519.

37 See Julie Cohen, *Between Truth and Power* (OUP 2019) 202ff (discussing global institutions).

38
*The Hollywood Reporter*, ‘MPA Sends Cease and Desist Letter to Instagram Over “Literally False and Highly Misleading” PG-13 Rating’ (2025) <www.hollywoodreporter.com/business/digital/mpa-cease-and-desist-instagram-pg-13-rating-1236418825/> accessed 25 November 2025.

39 An earlier limited concept note has been previously described in Martin Husovec, ‘Trusted Content Creators’ (LSE Law, 1 December 2022) Policy Briefing Paper No 52 <https://ssrn.com/abstract=4290917> accessed 25 November 2025.

40 See Martin Husovec, ‘Rising Above Liability: The Digital Services Act as a Blueprint for the Second Generation of Global Internet Rules’ (2023) 38(3) Berkeley Technology Law Journal 884; Jeff Kosseff, *The Twenty-Six Words that Created the Internet* (Cornell UP 2019).

41 Commission, ‘Illegal and Harmful Content on the Internet’ (Communication) COM (96) 487 final, 13.

42 Recommendation CM/Rec(2011)7 of the Committee of Ministers to member States on a new notion of media (21 September 2011).

43 Most recently, the ECtHR solidified this framing in *Google v Russia* App no 37027/22 (ECtHR, 8 July 2025), para 79. The ECtHR’s reference to duties of care and due diligence is an obvious reference to the approaches of the UK and EU in the national legislation.

44 Recommendation CM/Rec(2011)7, para 15.

45 Recommendation CM/Rec(2018)2 of the Committee of Ministers to member States on the roles and responsibilities of Internet intermediaries (7 March 2018).

46 ibid para 1.3.9.

47 Commission Recommendation (EU) 2018/334 of 1 March 2018 on measures to effectively tackle illegal content online [2018] OJ L63/50.

48 See *Online Harms* (White Paper, CP 57, 2019) <https://assets.publishing.service.gov.uk/media/605e60c6e90e07750810b439/Online_Harms_White_Paper_V2.pdf> accessed 25 November 2025; for the legislative history, see also <https://commonslibrary.parliament.uk/research-briefings/cbp-9579/> accessed 25 November 2025.

49 Husovec (n 40).

50 Convention for the Protection of Human Rights and Fundamental Freedoms (European Convention on Human Rights, as amended) (ECHR), art 10.

51 Husovec (n 40) 909.

52 See Council of Europe, ‘Recommendation of the Committee of Ministers to Member States on Online Safety and User Empowerment’ (December 2025) <www.coe.int/en/web/freedom-expression/committee-of-experts-online-safety-and-empowerment-of-content-creators-and-users-msi-esec> accessed 25 November 2025 (text on file with the author, who is one of the co-rapporteurs of the recommendation).

53 Amanda Reid, Lorcan Neill and Evan Ringel, ‘Nerd Harder: A Typology of Techno-Legal Solutionist Logics in Child Online Safety Laws’ (2025) 17(3) Policy & Internet.

54 Marshall McLuhan, *Understanding Media: The Extensions of Man* (McGraw-Hill 1964); Lessig (n 13).

55 See Renée DiResta, *Invisible Rulers: The People Who Turn Lies into Reality* (Public Affairs 2024).

56 NPR, ‘Here Are 4 Key Points from the Facebook Whistleblower’s Testimony on Capitol Hill’ (2021) <www.npr.org/2021/10/05/1043377310/facebook-whistleblower-frances-haugen-congress> accessed 25 November 2025.

57 Benkler (n 5) 31.

58 Recommendation on Online Safety and User Empowerment (n 52) para 67.

59 Natali Helberger, Jo Pierson and Thomas Poell, ‘Governing Online Platforms: From Contested to Cooperative Responsibility’ (2018) 34(1) The Information Society 1; Benjamin Farrand, ‘Co-regulating Principles for System Safety: Agency by Design’ (2025) 59 Computer Law & Security Review; Helberger (n 16) 849.

60 Douglass C North, *Institutions, Institutional Change and Economic Performance* (CUP 1990).

61 Daron Acemoglu and James A Robinson, *Why Nations Fail* (Crown 2012).

62 Elinor Ostrom, ‘Beyond Markets and States: Polycentric Governance of Complex Economic Systems’ (2010) 100 American Economic Review 641 (emphasis added).

63 Francis Fukuyama, *The Origins of Political Order* (Farrar, Straus & Giroux 2012); Francis Fukuyama, *Trust: The Social Virtues and Creation of Prosperity* (Free Press 1995) 23ff.

64 Andrew Murray, *The Regulation of Cyberspace: Control in the Online Environment* (Routledge 2007) 233–51.

65 Online Safety Act 2023 (the act does not repeal the UK implementation of E-Commerce Regulations 2002, and implicitly builds upon them in some sections, eg s 71); Regulation (EU) 2022/2065 of the European Parliament and of the Council of 19 October 2022 on a Single Market For Digital Services and amending Directive 2000/31/EC (Digital Services Act) [2022] OJ L277/1, Recital 41; see also *Zalando SE v European Commission* (T-348/23) [2025] ECLI:EU:T:2025:821, para 35).

66 See DSA, art 3(g) and (i); OSA, s 3.

67 See DSA, art 3(j); OSA, s 3.

68 Under DSA, art 19, generous carve-outs exist for design obligations of online platforms. Any company that is micro and small is not subject to such obligations, unless it qualifies as a VLOP. There are no such carve-outs under the OSA, even though obligations are meant to be proportionate (see eg OSA, ss 10(10), 13(1), 16(1), 17(6), 19(9), 27(9), 30(1) and 74(7)).

69 Under the DSA, VLOPs and VLOSEs require 45 million monthly active users in the EU (see DSA, art 33(1)). Under the OSA, Ofcom defines the thresholds for Category 1 and 2A services, following a procedure foreseen by the OSA. The draft laid by Ofcom has been confirmed by both Houses of Parliament, resulting in The Online Safety Act 2023 (Category 1, Category 2A and Category 2B Threshold Conditions) Regulations 2025. The Regulations define Category 1 service as a user-to-user service that either (a) has more than 34 million UK users if it includes a content recommender system or (b) has more than 7 million UK users if it includes a content recommender system, and functionality to share and forward regulated user-generated content. Category 2A service is a generalist search engine that has more than 7 million UK users.

70 European Commission, ‘Guidelines for Providers of VLOPs and VLOSEs on the Mitigation of Systemic Risks for Electoral Processes’ (2024).

71 European Commission, ‘Guidelines on Measures to Ensure a High Level of Privacy, Safety and Security for Minors Online, Pursuant to Article 28(4) of Regulation (EU) 2022/2065’ (2025) <https://digital-strategy.ec.europa.eu/en/library/commission-publishes-guidelines-protection-minors> accessed 25 November 2025.

72 See DSA, arts 30–2.

73 The European Commission adopted the delegated acts on auditing (2023), on transparency reporting (2024) and on data access (2025). While the UK’s OSA did not include any data access provisions, they were recently introduced in the Data (Use and Access) Act 2025, s 125, which inserted a new s 154A into the OSA.

74 On the private and collective enforcement, see Martin Husovec, *Principles of the Digital Services Act* (OUP 2024) 417ff.

75 DSA, art 22.

76 ibid art 86.

77 ibid art 40.

78 ibid art 37.

79 ibid art 21.

80 See Husovec, *Principles of the Digital Services Act* (n 74) 256.

81
*Google v Russia* (n 43) para 90ff.

82 DSA, art 14(1); see the discussion on art 14 in Benjamin Raue and Franz Hofmann (eds), *Digital Services Act: Article-by-Article Commentary* (Bloomsbury 2024).

83 OSA, s 18.

84 Regulation (EU) 2024/1083 of the European Parliament and of the Council of 11 April 2024 establishing a common framework for media services in the internal market and amending Directive 2010/13/EU (European Media Freedom Act) [2024] OJ L1083, art 18.

85 See DSA, art 14(4); see also the discussion by Husovec, *Principles of the Digital Services Act* (n 74) 253ff; Raue and Hofmann (n 82) 280 [art 14, para 19].

86 OSA, ss 18(3) (advance notice) and 18(4)–(5) (exemptions).

87 European Media Freedom Act, art 18(1).

88 ibid art 17(4).

89 ibid.

90 See Yiqing Hua and others, ‘Generative AI in User-Generated Content’ [2024] Extended Abstracts of the CHI Conference on Human Factors in Computing Systems art 471.

91 See ‘Social Media Creators to Overtake Traditional Media in Ad Revenue This Year’ *The Guardian* (10 June 2025) <www.theguardian.com/media/2025/jun/10/social-media-creators-to-overtake-traditional-media-in-ad-revenue-this-year> accessed 25 November 2025.

92 See ‘Wikipedia Bans Daily Mail as “Unreliable” Source’ *The Guardian* (8 February 2017) <www.theguardian.com/technology/2017/feb/08/wikipedia-bans-daily-mail-as-unreliable-source-for-website> accessed 25 November 2025.

93 OSA, s 15(10).

94 ibid s 16(3)–(5).

95 DSA, art 37.

96 See European Commission, ‘Guidelines on the Protection of Minors’ (n 71) para 57.

97 See European Commission, ‘Commission Guidelines for Providers of Very Large Online Platforms and Very Large Online Search Engines on the Mitigation of Systemic Risks for Electoral Processes Pursuant to Article 35(3) of Regulation (EU) 2022/2065’ [2024] OJ C2024/03014, para 27(d).

98 See European Commission, ‘Guidelines on the Protection of Minors’ (n 71) para 65(f).

99 See European Commission, ‘Guidelines on Electoral Processes’ (n 97) para 27(d).

100 Francis Fukuyama and others, ‘Middleware for Dominant Digital Platforms: A Technological Solution to a Threat to Democracy’ (Freeman Spogli Institute 2020) <https://fsi-live.s3.us-west-1.amazonaws.com/s3fs-public/cpc-middleware_ff_v2.pdf> accessed 25 November 2025; Luke Hogg and others, ‘Shaping the Future of Social Media with Middleware’ (2025) <https://arxiv.org/pdf/2412.10283> accessed 25 November 2025; Daphne Keller, ‘The Future of Platform Power: Making Middleware Work’ (2021) 32 Journal of Democracy 168 <www.journalofdemocracy.org/articles/the-future-of-platform-power-making-middleware-work/> accessed 25 November 2025; Fiona M Scott Morton, ‘It’s Time for the European Union to Rethink Personal Social Networking’ (Bruegel 2024) <www.bruegel.org/policy-brief/its-time-european-union-rethink-personal-social-networking> accessed 25 November 2025; Francisco De Abreu Duarte, ‘Digital Constitutionalism and Online Content Moderation: Three Models for the Future of Online Speech’ in Giovanni De Gregorio and others (eds), *The Oxford Handbook of Digital Constitutionalism* (OUP 2025).

101 Recommendation on Online Safety and User Empowerment (n 52) para 73.

102 See Fiona M Scott Morton and others, ‘Equitable Interoperability: The “Supertool” of Digital Platform Governance’ (2025) 40(3) Yale J on Reg 1013.

103 The middleware is being considered under the Recommendation for Online Safety and User Empowerment (n 52). Various vertical and horizontal interoperability options are being discussed as part of the review of art 7 of the EU Digital Markets Act.

104 Habermas, *A New Structural Transformation* (n 1) 63.

105 On the quantitative and qualitative description of Bluesky third-party labelling, see Leonhard Balduf and others, ‘Looking at the Blue Skies of Bluesky’ (2024) <https://arxiv.org/abs/2408.12449> accessed 25 November 2025.

106 Quality assurance relied on reputation embedded in social networks—what sociologists like Mark Granovetter later described as embedded exchange, see Mark Granovetter, ‘Economic Action and Social Structure: The Problem of Embeddedness’ (1985) 91(3) American Journal of Sociology 481.

107 See Susan Strasser, *Satisfaction Guaranteed: The Making of the American Mass Market* (Pantheon Books 1989) 30ff.

108 ibid 88.

109 Richard Posner, *Economic Analysis of Law* (9th edn, Aspen Publishing 2014) §3.4: 43–5.

110 Recommendation on Online Safety and User Empowerment (n 52) para 72.

111 Regulation (EU) 2017/1001 of the European Parliament and of the Council of 14 June 2017 on the European Union trade mark [2017] OJ L154/1, art 83(1); Trade Marks Act 1994, s 50.

112 ibid.

113 See Xiomara F Quiñones-Ruiz and others, ‘Why Early Collective Action Pays Off: Evidence from Setting Protected Geographical Indications’ (2016) 1(2) Renewable Agriculture and Food Systems 1.

114 In theory, and especially for jurisdictions with weaker public institutions, the system could also work without state certification by relying on platforms and users as a check on their potential abuse.

115 See Britannica, ‘Collective Action Problem’ <www.britannica.com/topic/collective-action-problem-1917157> accessed 25 November 2025.

116 DSA, art 23.

117 Husovec, *Principles of the Digital Services Act* (n 74) 195.

118 See DSA, art 14(4); for discussions in the literature, see João Pedro Quintais, Naomi Appelman and Ronan Ó Fathaigh, ‘Using Terms and Conditions to Apply Fundamental Rights to Content Moderation’ (2023) 24(5) German Law Journal 881; Husovec, *Principles of the Digital Services Act* (n 74) 249ff; Folkert Wilman, Sualius L Kaléda and Paul-John Loewenthal, *The EU Digital Services Act: A Commentary* (OUP 2024) art 14; Raue and Hofmann (n 82) art 14.

119 See Eli Pariser, *The Filter Bubble: What the Internet Is Hiding from You* (Penguin Books 2012); Cass R Sunstein, *Republic.com* (Princeton UP 2002); Axel Bruns, *Are Filter Bubbles Real?* (Polity Press 2019).

120 Aleksandra Kuczerawy and Joao Quintais, ‘“Must-Carry”, Special Treatment and Freedom of Expression on Online Platforms: A European Story’ [2025] European Law Open 1.

121
*Google v Russia* (n 43) paras 74ff.

122 Baker (n 2) 16.

123 ibid 7.

124 Helberger (n 16).

125 Couldry (n 13) 58.

126
*Handyside v United Kingdom* App no 5493/72 (ECtHR, 7 December 1976), para 49.

127 Josiah Ober, ‘The Original Meaning of “Democracy”: Capacity to Do Things, Not Majority Rule’ (Princeton/Stanford Working Papers in Classics 2007) Paper no 090704 <https://ssrn.com/abstract=1024775> accessed 25 November 2025.

128 Hayek (n 36).

129 Decision, EY and WIK Consult, ‘Study of Interoperability Tools in the Digital Single Market Workshop’ (slide presentation, 4 November 2025) 31 (on file with the author).

130 Friederike Stock and others, ‘People in 26 Countries Prefer Individual Control to Commercial or Governmental Control over Online Choice Architectures’ <osf.io/haqu9_v1> accessed 25 November 2025.

131 Ostrom (n 62).

132 Randall Munroe, ‘Duty Calls’ (xkcd, 12 October 2008) <https://xkcd.com/386> accessed 25 November 2025.

